# Clinical and biochemical factors associated with insulin therapy in gestational diabetes: The role of OGTT‐based groups

**DOI:** 10.1113/EP093778

**Published:** 2026-08-10

**Authors:** Libera Troìa, Martina Garassino, Riccardo Bertinato, Caroline Leitao Thomaz, Giulia Turatello, Chiara Airoldi, Alessandro Libretti, Daniela Surico, Valentino Remorgida

**Affiliations:** ^1^ Department of Gynaecology and Obstetrics University Hospital Maggiore della Carità Novara Italy; ^2^ Department of Translational Medicine University of Eastern Piedmont Novara Italy

**Keywords:** antenatal care, GDM, gestational diabetes, insulin therapy, nutritional therapy, OGTT, phenotypes

## Abstract

Gestational diabetes mellitus (GDM) represents one of the most common metabolic disorders of pregnancy. This study aimed to identify clinical and biochemical factors associated with antenatal insulin therapy, focusing on the association with oral glucose tolerance test (OGTT)‐based phenotypes. Retrospective observational study among GDM women, divided according to their treatment (nutritional therapy (NT) or insulin therapy). GDM was classified into three phenotypes based on OGTT results: isolated fasting hyperglycaemia (IFH‐GDM), isolated post‐load hyperglycaemia (IPH‐GDM) and combined hyperglycaemia (CH‐GDM). Maternal demographic, obstetric and biochemical data were analysed. Multivariable logistic regression analysis was performed to identify factors independently associated with insulin therapy. Among 458 women, 39.3% required insulin therapy. Independent factors associated with the use of insulin therapy included pre‐pregnancy obesity (odds ratio (OR) 2.07, 95% CI: 1.21–3.55), spontaneous miscarriage history (OR 1.89, 95% CI: 1.21–2.93), pregestational hypothyroidism (OR 2.63, 95% CI: 1.08–6.42), GDM diagnosis at 24–28 weeks versus earlier diagnosis (OR 0.45, 95% CI: 0.28–0.72), and GDM phenotype. Compared with IFH‐GDM, IPH‐GDM was associated with a lower risk of insulin therapy (OR 0.51, 95% CI: 0.31–0.84), whereas CH‐GDM was associated with a higher risk (OR 1.90, 95% CI: 1.10–3.31). In conclusion, women with fasting and combined hyperglycaemia represent the highest‐risk group for insulin therapy. Integrating OGTT‐based phenotypes with clinical risk factors was associated with increased likelihood of insulin therapy requirement and may support individualized clinical assessment.

## Abstract (highlights)

- **What is the central question of this study?**
  Which clinical and biochemical factors – particularly OGTT‐based gestational diabetes phenotypes – are associated with the need for antenatal insulin therapy in women with gestational diabetes mellitus?
- **What is the main finding and its importance?**
  Fasting and combined hyperglycaemia phenotypes, obesity, miscarriage history, hypothyroidism and early diagnosis are independently associated with the use of insulin therapy. Identifying these risks early enables personalized monitoring and treatment, improving glycaemic control and potentially reducing maternal and neonatal complications.

## 1 INTRODUCTION

Gestational diabetes mellitus (GDM) represents one of the most common metabolic disorders of pregnancy and is associated with a wide range of adverse perinatal outcomes, including macrosomia, shoulder dystocia, caesarean section and neonatal hypoglycaemia (Farahvar et al., 2019). In addition to these short‐term complications, GDM confers a significant long‐term burden, being strongly linked to maternal glucose metabolism disorders after delivery and to an increased risk of adiposity in offspring (Sweeting et al., 2022).

Appropriate diagnosis and management of GDM is crucial to mitigate both maternal and neonatal adverse consequences. Lifestyle and nutritional therapy (NT) constitute the cornerstone of treatment; however, when glycaemic targets are not achieved, pharmacological therapy must be introduced (Care & Suppl, 2022). Early identification of women at higher risk of requiring insulin therapy could allow tailored follow‐up and preventive strategies, potentially improving pregnancy and neonatal outcomes.

Several studies have investigated predictors of NT failure, highlighting clinical, biochemical and even genetic determinants (Alvarez‐Silvares et al., 2022; Barnes et al., 2016; Dincgez et al., 2023; Ducarme et al., 2019; Sweeting et al., 2022). Among clinical risk factors, advanced maternal age, elevated pre‐pregnancy body mass index (BMI), previous history of GDM, prior macrosomic birth, early diagnosis (<24 weeks), hypothyroidism, assisted reproductive technology, family history of diabetes, and South Asian ethnicity have been involved (Ford et al., 2022; Mecacci et al., 2021; Morikawa et al., 2021; Rosinha et al., 2022; Topaloglu et al., 2023; Wang et al., 2021). Biochemical markers could provide additional predictive value. Elevated fasting plasma glucose, HbA1c at diagnosis, abnormal 1‐h oral glucose tolerance test (OGTT), and the number of pathological values at OGTT have in fact emerged as independent factors associated with the use of insulin therapy (Akinci et al., 2008; Barnes et al., 2016; Troìa et al., 2024; Wong & Jalaludin, 2011).

GDM can be operationally stratified into three groups based on OGTT glucose patterns, commonly referred to in the literature as phenotypes: (1) isolated fasting hyperglycaemia (IFH‐GDM), (2) isolated post‐load hyperglycaemia (IPH‐GDM), and (3) combined fasting and post‐load hyperglycaemia (CH‐GDM). These phenotypes differ not only in metabolic features but also in maternal and fetal outcomes (Chatzakis et al., 2022, 2023).

Despite extensive research, the factors associated with insulin therapy requirement in women with GDM remain incompletely characterized, particularly regarding the role of OGTT glucose patterns. Therefore, the aim of this study was to identify clinical and biochemical factors associated with insulin therapy in women with GDM, comparing those successfully managed with NT alone to those requiring pharmacological intervention. Among biochemical factors, we examined whether OGTT patterns are associated with subsequent inadequate glycaemic control.

## 2 METHODS

The study was conducted in accordance with the *Declaration of Helsinki*, and Institutional Review Board approval was obtained (Prot. 753/CE). Written informed consent was obtained from all participants.

This was a retrospective observational study conducted at the Maternal Fetal Unit of the Department of Gynecology and Obstetrics, University Hospital Maggiore della Carità in Novara, Piedmont, Italy, from January 2022 to February 2024.

According to international guidelines, a 75‐g oral glucose tolerance test was administered as GDM screening in women with risk factors (≥35 years, BMI >25 kg/m^2^, family history of diabetes, previous macrosomia, or high‐risk ethnicity) between 24 and 28 weeks of gestation. Early screening at 16–18 weeks was implemented in high‐risk women (previous GDM, BMI greater than 30 kg/m^2^, fasting plasma glucose between 100–125 mg/dL at first trimester blood test), and repeated at 24–28 weeks if negative (Standard italiani per la cura del diabete mellito, 2018). GDM phenotypes were defined according to OGTT glucose values using the IADPSG criteria (fasting ≥92 mg/dL, 1‐h ≥180 mg/dL, 2‐h ≥153 mg/dL) (Metzgerer et al., 2010). Based on these thresholds, patients were classified as follows: (1) isolated fasting hyperglycaemia (IFH‐GDM), defined by an abnormal fasting glucose value with normal 1‐ and 2‐h values; (2) isolated post‐load hyperglycaemia (IPH‐GDM), defined by normal fasting glucose and at least one abnormal post‐load value (1 h and/or 2 h); and (3) combined hyperglycaemia (CH‐GDM), defined by abnormal fasting glucose in combination with at least one abnormal post‐load value. According to these criteria, abnormal glucose values were defined as fasting plasma glucose ≥92 mg/dL, 1 h ≥180 mg/dL, and 2 h ≥153 mg/dL.

After diagnosis, patients were instructed on self‐monitoring of blood glucose, measuring capillary blood sugar 4 times a day: fasting and 1 h after meals. Nutrition counselling and an individualized diet plan were provided to control gestational weight gain (GWG) and maintain a normoglycaemic state. Lifestyle interventions were also discussed and recommended in each consultation.

In accordance to the Italian National Guidelines, the target glycaemic levels were the following: ≤90 mg/dL fasting and ≤130 mg/dL at 1 h and ≤120 mg/dL at 2 h after meals (Standard italiani per la cura del diabete mellito, 2018). If more than half of the blood glucose measurements exceeded the target during the first 2‐weeks of assessment, insulin treatment was recommended (Standard italiani per la cura del diabete mellito, 2018). To normalize fasting hyperglycaemia, long‐acting insulin analogue was the starting therapy in the evening; if also the postprandial values were impaired, a rapid insulin analogue was added.

Women were divided into two groups according to the treatment needed to achieve adequate glycaemic control: NT group and Insulin group. The most intensive treatment required during pregnancy was considered when categorizing each patient (i.e., patients requiring insulin at any time during pregnancy were classified in the insulin group. De‐escalation from insulin therapy to NT alone is not standard clinical practice in GDM management and was not observed in our cohort. We collected maternal characteristics (age, ethnicity, GWG, BMI before pregnancy and at delivery, smoking status, family history of diabetes, pregestational maternal diseases), obstetric history (mode of conception, parity, previous or current pregnancy complications), delivery and neonatal outcome (gestational age at delivery, mode of delivery, APGAR score at the first and fifth minutes, neonatal intensive care unit (NICU) admission, sex, birthweight, fetal growth patterns). Biochemical parameters (fasting glucose at first trimester, OGTT values, glycated haemoglobin at diagnosis) were also evaluated.

Using the levels of the fasting, 1‐ and 2‐h values after 75 g OGTT, three phenotypes of GDM were considered: isolated fasting hyperglycaemia (IFH‐GDM), isolated post‐load hyperglycaemia (IPH‐GDM) and combined hyperglycaemia (CH‐GDM).

The exclusion criteria were: pre‐gestational diabetes mellitus (already known or diagnosed in the first trimester of pregnancy by a fasting glucose level ≥126 mg/dL or glycated haemoglobin 6.5% and reconfirmed on a subsequent testing), twin pregnancies, asthma, chronic hypertension, autoimmune diseases, nephropathy, current use of corticosteroid, and fetal malformations. Institutional Review Board approval was obtained with the protocol number Prot. 753/CE.

### 2.1 Statistical analysis

Descriptive statistics were reported for the overall sample and stratified by treatment group (NT vs. insulin therapy). Categorical variables were expressed as absolute frequencies and percentages, while continuous variables were presented as means ± standard deviation or medians and interquartile range (IQR), as appropriate based on distribution.

Between‐group comparisons were performed using the chi‐square or Fisher's exact test for categorical variables and Student's *t*‐test or the Mann–Whitney *U*‐test for continuous variables, depending on data distribution assessed by visual inspection and normality testing.

To identify factors independently associated with insulin therapy, a multivariable logistic regression model was constructed. Variables with a *P*‐value < 0.05 in univariate analysis were initially included, along with clinically relevant variables. Covariates included in the multivariable model were selected a priori based on clinical relevance, subject‐matter knowledge, and previously reported associations with insulin therapy requirement in GDM, rather than through automated stepwise selection procedures. We specifically considered potential confounding structures when selecting covariates and avoided including variables that could plausibly lie on the causal pathway between baseline maternal characteristics and insulin therapy requirement. Odds ratios (ORs) with 95% confidence intervals (95% CI) were reported.

Model assumptions were assessed as follows: linearity of continuous variables with respect to the log‐odds was evaluated using graphical methods; multicollinearity was assessed using variance inflation factors (VIF), with a threshold of >5 indicating potential collinearity; and model fit was evaluated using standard goodness‐of‐fit measures.

Missing data were handled using complete‐case analysis, as the proportion of missing values was low.

No formal adjustment for multiple comparisons was performed; therefore, the results should be interpreted as exploratory.

Statistical significance was set at *P* < 0.05 (two‐tailed). All analyses were performed using SAS version 9.4 (SAS Institute Inc., Cary, NC, USA) and R version 4.4.1 (R Foundation for Statistical Computing, Vienna, Austria). Statistical modelling was conducted using standard regression modelling packages available within the R environment.

## 3 RESULTS

The study population included 458 women, stratified into two groups according to the treatment required to achieve the adequate glycaemic control: 278 (60.7%) in the NT group and 180 (39.3%) in the insulin group.

Most participants were Caucasian (*n* = 306, 66.8%). Only a minority reported smoking (*n* = 24, 5.3%), while the vast majority conceived spontaneously (*n* = 429, 93.7%). The mean maternal age was 33.5 years (SD 5.37; range 19–46 years), and the mean pre‐pregnancy BMI was 26.6 kg/m^2^ (SD 5.61) (Table 1).

**TABLE 1:** Demographic and clinical characteristics of the study population.

	All (*N* = 458)	NT group (*n* = 278)	Insulin group (*n* = 180)	*P*
**Caucasian ethnicity**	306 (66.81)	207 (74.46)	99 (55.00)	**<0.0001**
**Age (years)**	33.50 ± 5.37	33.22 ± 5.35	33.95 ± 5.38	0.1533
≥35 years	210 (45.85)	119 (42.81)	91 (50.56)	0.1040
≥40 years	63 (13.76)	37 (13.31)	26 (14.44)	0.7305
**Smoking habit**				
Yes	24 (5.25)	14 (5.04)	10 (5.59)	0.7967
**Parity**				
Nulliparous	170 (37.12)	119 (42.81)	51 (28.33)	**0.0017**
**History of spontaneous miscarriage**				
Yes	167 (36.46)	88 (31.65)	79 (43.89)	**0.0079**
**Onset of pregnancy**				
Spontaneous	429 (93.67)	259 (93.17)	170 (94.44)	0.5830
**BMI (kg/m^2^)**	26.60 ± 5.61	25.51 ± 5.34	28.28 ± 5.63	**<0.0001**
Normal weight (<25 kg/m^2^)	202 (44.10)	145 (52.16)	57 (31.67)	**<0.0001**
Overweight (BMI 25–30 kg/m^2^)	136 (29.69)	77 (27.70)	59 (32.78)	
Obese (≥30 kg/m^2^)	120 (26.20)	56 (20.14)	64 (35.56)	
**Family history of diabetes mellitus**				
Yes	205 (44.76)	113 (40.65)	92 (51.11)	**0.0278**
**Clinical characteristics**				
History of GDM	83 (18.12)	34 (12.23)	49 (27.22)	**<0.0001**
Previous macrosomia	34 (7.42)	10 (3.60)	24 (13.33)	**0.0001**
Pre‐gestational hypothyroidism	30 (6.55)	12 (4.32)	18 (10.00)	**0.0163**
Previous HDP in pregnancy	12 (2.62)	6 (2.16)	6 (3.33)	0.4419
Previous PPH	8 (1.75)	5 (1.80)	3 (1.67)	0.9162
Previous caesarean section	88 (19.21)	45 (16.19)	43 (23.89)	**0.0410**
Psychiatric disorders	10 (2.18)	3 (1.08)	7 (3.89)	0.0542

*Note*: Data are expressed as means ± standard deviation or as absolute number (percentage). *P* < 0.05 was considered statistically significant (marked in bold).

Abbreviations: BMI, body mass index; GDM, gestational diabetes mellitus; HDP, hypertensive disorders of pregnancy; NT, nutritional therapy; PPH, postpartum haemorrhage.

No significant differences were observed between the groups with respect to maternal age, mode of conception or smoking status (Table 1). However, women in the NT group were more frequently Italian (74.5% vs. 55.0%, *P *< 0.0001) and nulliparous (42.8% vs. 28.3%, *P* = 0.0017). Women requiring insulin therapy had a significantly higher pre‐pregnancy BMI (28.28 ± 5.63 vs. 25.51 ± 5.34 kg/m^2^, *P *< 0.0001) and a greater prevalence of obesity (BMI ≥30 kg/m^2^: 35.6% vs. 20.1%, *P* = 0.0001). In addition, they reported more often a family history of diabetes mellitus (51.1% vs. 39.2%, *P* = 0.0132), a previous pregnancy complicated by GDM (27.2% vs. 11.5%, *P *< 0.0001), a history of macrosomia (13.3% vs. 3.6%, *P* = 0.0001) and previous spontaneous miscarriage (43.9% vs. 31.7%, *P* = 0.0079) (Table 1). The demographic and clinical characteristics of the two groups are summarized in Table 1.

Approximately 32% of women presented with high‐risk factors for GDM and therefore underwent OGTT at 16–18 weeks; among these, 83% tested positive. Early OGTT was more frequently performed in women who subsequently required insulin therapy (45.6% vs. 24.1%, *P *< 0.001), with a higher proportion of positive results (90.2% vs. 74.6%, *P* = 0.0112).

First‐trimester fasting blood glucose levels were significantly higher in the insulin group compared with the NT group (95.06 ± 19.80 vs. 82.20 ± 14.00 mg/dL, *P* = 0.0001), whereas glycated haemoglobin measured at the time of GDM diagnosis did not differ significantly between groups (5.51% ± 0.48% vs. 5.26% ± 0.16%, *P* = 0.0927) (Table 2).

**TABLE 2:** Biochemical features: OGTT characteristics and classification of GDM phenotypes.

	All (*N* = 458)	NT group (*n* = 278)	Insulin group (*n* = 180)	*P*
**Fasting blood sugar in the first trimester (mg/dL)**	87.89 ± 17.93	82.20 ± 14.00	95.06 ± 19.80	**0.0001**
**HbA1c at diagnosis of GDM (%)**	5.44 ± 0.43	5.26 ± 0.16	5.51 ± 0.48	0.0927
**OGTT performed at 16–18** **weeks**				
Not assessed	309 (67.47)	211 (75.90)	98 (54.44)	**<0.0001**
Negative	25 (5.46)	17 (6.12)	8 (4.44)	
IFH‐GDM	66 (14.41)	26 (9.35)	40 (22.22)	
IPH‐GDM	30 (6.55)	15 (5.40)	15 (8.33)	
CH‐GDM	28 (6.11)	9 (3.24)	19 (10.56)	
**OGTT performed at 24–28** **weeks**				
Not assessed	147 (32.10)	65 (23.38)	82 (45.56)	**<0.0001**
Negative				
IFH‐GDM	124 (27.07)	82 (29.50)	42 (23.33)	
IPH‐GDM	126 (27.51)	103 (37.05)	23 (12.78)	
CH‐GDM	61 (13.32)	28 (10.07)	33 (18.33)	
**Time of GDM diagnosis**	*N* = 431	*N* = 261	*N* = 170	
16–18 weeks	124 (28.77)	50 (19.16)	74 (43.53)	**<0.0001**
24–28 weeks	307 (71.23)	211 (80.84)	96 (56.47)	
**GDM phenotype** ^**^				
IFH‐GDM	189 (43.85)	108 (41.38)	81 (47.65)	**<0.0001**
IPH‐GDM	155 (35.96)	117 (44.83)	38 (22.35)	
CH‐GDM	87 (20.19)	36 (13.79)	51 (30.00)	
**OGTT: value (mg/dL)**				
I value	93.14 ± 11.39	90.21 ± 9.10	97.67 ± 13.01	**<0.0001**
II value	166.48 ± 33.79	164.36 ± 32.41	169.85 ± 35.73	0.1192
III value	139.96 ± 33.67	137.21 ± 29.65	144.32 ± 38.91	0.0577
**OGTT: number of altered value**	*N* = 385	*N* = 236	*N* = 149	
1	256 (66.49)	174 (73.73)	82 (55.03)	**<0.0001**
2	98 (25.45)	55 (23.31)	43 (28.86)	
3	31 (8.05)	7 (2.97)	24 (16.11)	

*Note*: Data are expressed as means ± standard deviation or as absolute number (percentage).

** Only positive OGTTs were considered for the analysis. This was regardless the gestational age at which they were performed (16–18 vs. 24–28 weeks). *P* < 0.05 was considered statistically significant (marked in bold).

Abbreviations: CH‐GDM, combined hyperglycaemia; GDM, gestational diabetes mellitus; IFH‐GDM, isolated fasting hyperglycaemia; IPH‐GDM, isolated post‐load hyperglycaemia; NT, nutritional therapy; OGTT, oral glucose tolerance test.

When considering GDM phenotype, CH‐GDM occurred more frequently in the Insulin group (30% vs. 13.79%, *P *< 0.001), while IPH‐GDM was more frequent in NT group (44.82% vs. 22.35%, *P *< 0.001) (Table 2).

OGTT fasting glucose levels were higher among women requiring insulin therapy (97.67 ± 13.01 vs. 90.21 ± 9.10 mg/dL, *P *< 0.001). Women in the NT group presented more often with a single abnormal value (73.7% vs. 55.0%, *P *< 0.001) (defined according to IADPSG thresholds), whereas those in the insulin group more frequently exhibited three abnormal values (16.1% vs. 3.0%, *P *< 0.001) (defined according to IADPSG thresholds) (Table 2). Detailed characteristics of OGTT performed at 16–18 weeks and at 24–28 weeks are provided in Table 1.

A multivariable logistic regression analysis was conducted, and the findings are presented in Table 3. A diagnosis of GDM at 24–28 weeks was associated with a significantly lower likelihood of requiring insulin therapy compared with an earlier diagnosis (OR 0.45 [95% CI: 0.28–0.72]). IPH‐GDM phenotype was linked to a lower likelihood of insulin treatment compared to the IFH‐GDM phenotype (OR 0.51 [95% CI: 0.31–0.84]). In contrast, women with CH‐GDM phenotype showed a numerically higher odds of NT failure compared with the IFH‐GDM group, although this association did not reach statistical significance (OR 1.90 [95% CI: 0.10–3.31]). Independent predictors of insulin requirement included ethnicity, pre‐pregnancy BMI, history of spontaneous miscarriage, and pregestational hypothyroidism (Table 3).

**TABLE 3:** Multivariable logistic regression analysis.

Variables	OR [95% CI]	*P*
**Ethnicity**		
Caucasian vs. others	0.53 [0.34; 0.83]	0.0057
**Spontaneous miscarriage**		
Yes vs. no	1.89 [1.21; 2.93]	0.0049
**BMI**		Overall *P* = 0.0301
Overweight vs. normal weight	1.40 [0.84; 2.33]	0.19
Obese vs. normal weight	2.07 [1.21; 3.55]	0.008
Obese vs. overweight	^*^ *1.48 [not estimable]* ^**^	
**Pre‐gestational hypothyroidism**		
Yes vs. no	2.63 [1.08; 6.42]	0.0335
**Time of GDM diagnosis**		
24–28 vs. 16–18 weeks	0.45 [0.28; 0.72]	0.0010
**GDM phenotype**		Overall *P *< 0.0001
IPH‐GDM vs. IFH‐GDM	0.51 [0.31; 0.84]	0.008
CH‐GDM vs. IFH‐GDM	1.90 [1.10; 3.31]	0.17
CH‐GDM vs. IPH‐GDM	^*^ *3.73 [not estimable]* ^**^	

* Additional pairwise odds ratios were algebraically re‐expressed from the fitted model contrasts already reported in the table.

** Confidence intervals and *P*‐values require the original model variance–covariance matrix and could not be reconstructed from the available aggregate output.

Abbreviations: BMI, body mass index; CH‐GDM, combined hyperglycaemia; GDM, gestational diabetes mellitus; IFH‐GDM, isolated fasting hyperglycaemia; IPH‐GDM, isolated post‐load hyperglycaemia.

Obstetric and delivery outcomes are presented in Table 4. BMI at delivery was higher for women on insulin therapy (31.06 ± 5.69 vs. 28.65 ± 5.03 kg/m^2^, *P *< 0.0001); however, GWG was lower (7.73 ± 5.78 vs. 8.46 ± 5.35 kg, *P* = 0.1659) (Table 3). Gestational age at delivery was significantly lower in women requiring insulin therapy (38.70 ± 1.63 vs. 39.13 ± 1.66 weeks, *P* = 0.0058), which is consistent with the higher frequency of labour induction observed in this group (41.3% vs. 28.7%, *P* = 0.0055) (Table 4). No statistically significant differences were found regarding mode of delivery, the use of intrapartum analgesia, or obstetric or post‐partum complications such us haemorrhage, perineal trauma and manual removal of placenta (Table 4).

**TABLE 4:** Obstetric and delivery outcomes.

	All (*N* = 458)	NT group (*n* = 278)	Insulin group (*n* = 180)	*P*
**Obstetric complications**				
HDP	27 (5.91)	16 (5.76)	11 (6.15)	0.8630
IUGR	7 (1.53)	5 (1.80)	2 (1.11)	0.5581
ICP	15 (3.28)	12 (4.32)	3 (1.67)	0.1197
pPROM	7 (1.53)	6 (2.17)	1 (0.56)	0.1708
Gestational hypothyroidism	22 (4.81)	16 (5.76)	6 (3.35)	0.2414
**BMI at delivery (kg/m^2^)**	29.59 ± 5.42	28.65 ± 5.03	31.06 ± 5.69	**<0.0001**
**Gestational weight gain (kg)**	8.17 ± 5.53	8.46 ± 5.35	7.73 ± 5.78	0.1659
**Gestational age at delivery (weeks)**	38.96 ± 1.66	39.13 ± 1.66	38.70 ± 1.63	**0.0058**
**Preterm births**				
<37 weeks	34 (7.42)	20 (7.19)	14 (7.78)	0.8160
<34 weeks	7 (1.53)	5 (1.80)	2 (1.11)	0.7094
**Induction of labour**	*N* = 454	*N* = 275		
Yes	153 (33.70)	79 (28.73)	74 (41.34)	**0.0055**
**Oxytocin augmentation**	*N* = 345	*N* = 216	*N* = 129	
Yes	76 (22.03)	47 (21.76)	29 (22.48)	0.8757
**PROM**				
Yes	142 (31.00)	91 (32.73)	51 (28.33)	0.3200
**Positive GBS vaginal–rectal swab**	*N* = 415	*N* = 257	*N* = 158	
Yes	50 (12.05)	26 (10.12)	24 (15.19)	0.1268
**Meconium‐stained amniotic fluid**				
Yes	49 (10.72)	37 (13.31)	12 (6.70)	**0.0259**
**Epidural analgesia**				
Yes	82 (17.94)	52 (18.71)	30 (16.76)	0.5968
**Mode of delivery**				
Spontaneous vaginal delivery	292 (63.76)	186 (66.91)	106 (58.89)	0.2116
Caesarean section	136 (29.69)	76 (27.34)	60 (33.33)	
Vacuum‐assisted delivery	30 (6.55)	16 (5.76)	14 (7.78)	
**Manual removal of placenta** *	*N* = 321	*N* = 202	*N* = 119	
Yes	12 (3.74)	9 (4.46)	3 (2.52)	0.3775
**Blood loss at delivery (mL)**	383.81 ± 311.78	371.76 ± 314.86	402.51 ± 306.86	0.3039
**Post‐partum haemorrhage**				
Yes	61 (13.32)	31 (11.15)	30 (16.67)	0.0897

*Note*: Data are expressed as means ± standard deviation or as absolute number (percentage).

* Only on spontaneous and vacuum‐assisted delivery. *P* < 0.05 was considered statistically significant (marked in bold).

Abbreviations: BMI, body mass index; GBS, Group B streptococcus; HDP, hypertensive disorders of pregnancy; ICP, intrahepatic cholestasis of pregnancy; IUGR, intrauterine growth restriction; NT, nutritional therapy; pPROM, preterm prelabour rupture of membranes; PROM, prelabour rupture of membranes.

Regarding neonatal outcomes, birthweight was higher in fetuses of mothers in the insulin group (3355 ± 566.32 vs. 3237.28 ± 508.98 g, *P* = 0.0202), as was the rate of macrosomia (11.11% vs. 3.24%, *P* = 0.0006). Consistently, fetal growth patterns revealed a higher percentage of large for gestational age (LGA) fetuses in the insulin‐treated group (20.11% vs. 7.55%, *P* = 0.0002). No statistically significant differences were found regarding fetal sex, APGAR score and NICU admission (Table 5). Similarly, the less favourable neonatal outcomes observed among insulin‐treated women should not be interpreted as caused by insulin treatment itself, but more likely as reflecting the greater severity of the underlying metabolic disturbance.

**TABLE 5:** Neonatal outcomes.

	All (*N* = 458)	NT group (*n* = 278)	Insulin group (*n* = 180)	*P*
**Newborn sex**				
Male	252 (55.02)	153 (55.04)	99 (55.00)	0.9940
**Birthweight (g)**	3283.93 ± 534.80	3237.28 ± 508.98	3355 ± 566.32	0.0202
**Macrosomia, ≥4000 g**				
Yes	29 (6.33)	9 (3.24)	20 (11.11)	**0.0007**
**Fetal growth pattern**				
AGA	360 (78.77)	231 (83.09)	129 (72.07)	**0.0002**
SGA	36 (7.88)	25 (8.99)	11 (6.15)	
LGA	57 (12.47)	21 (7.55)	36 (20.11)	
IUGR	4 (0.88)	1 (0.36)	3 (1.68)	
**APGAR 1st min**	8.31 ± 1.55	8.34 ± 1.47	8.26 ± 1.66	0.5968
**APGAR 1st min <7**	39 (8.52)	22 (7.91)	17 (9.44)	0.5665
**APGAR 5th min**	8.88 ± 0.80	8.92 ± 0.67	8.83 ± 0.97	0.2819
**APGAR 5th min** **<7**	7 (1.53)	4 (1.44)	3 (1.67)	>0.999
**NICU admission**				
Yes	68 (14.85)	40 (14.39)	28 (15.56)	0.7315

*Note*: Data are expressed as means ± standard deviation or as absolute number (percentage). *P* < 0.05 was considered statistically significant (marked in bold). Abbreviations: AGA, appropriate for gestational age; IUGR, intrauterine growth restriction; LGA, large for gestational age; NICU, neonatal intensive care unit; SGA, small for gestational age.

## 4 DISCUSSION

Our study identified several clinical, biochemical and metabolic factors independently associated with the use of insulin therapy among women with GDM. In particular, the role of GDM phenotype emerged as central in determining the likelihood of NT failure. Although the term ‘phenotype’ is widely used in the literature to describe OGTT‐based classifications, these categories represent operational groupings based on glucose patterns rather than distinct pathophysiological entities. Therefore, they should be interpreted as clinically useful stratifications rather than true biological phenotypes.

Our findings indicate that women with IFH‐GDM and, even more markedly, those with CH‐GDM have a significantly higher probability of requiring insulin therapy. These findings may reflect different OGTT glucose patterns and degrees of metabolic disturbance among women with GDM, rather than distinct underlying pathophysiological entities (Chatzakis et al., 2023; Papachatzopoulou et al., 2020).

Fasting hyperglycaemia has been associated with greater degrees of hepatic glucose dysregulation and insulin resistance in observational studies (Papachatzopoulou et al., 2020). This pattern has been associated with higher rates of fetal overgrowth, including LGA, potentially related to maternal glycaemic exposure (Imoh et al., 2016).

In the combined phenotype (CH‐GDM), in addition to the hepatic resistance, a more pronounced alteration in glucose metabolism may be present at both muscular and adipose tissue levels, resulting in a global alteration of glucose and lipid metabolism (Chatzakis et al., 2023; Kotzaeridi et al., 2021). These patterns may also be consistent with impaired insulin secretion, which would impair the suppression of hepatic glucose production, leading to abnormal fasting blood glucose and increased plasma glucose after the load. These women may be less likely to achieve adequate glycaemic control with lifestyle interventions alone (Bird & Hawley, 2017; Papachatzopoulou et al., 2020).

Conversely, women with IPH‐GDM show a post‐load glucose pattern that may be more amenable to improvement with dietary and lifestyle measures, although progression to insulin therapy is not inevitable in any OGTT‐based group and depends on the overall clinical and metabolic context (Chatzakis et al., 2023).

Nutritional and lifestyle interventions can improve postprandial glycaemic control by modifying carbohydrate quantity and quality, meal timing and physical activity patterns (Allehdan et al., 2019; Ch'ng et al., 2019; Chatzakis et al., 2022). For this reason, lifestyle management remains a central component of treatment across all OGTT‐based categories, and early optimization of diet and GWG may reduce the likelihood of pharmacological escalation in some women (Ch'ng et al., 2019; Chatzakis et al., 2022). Accordingly, the need for insulin therapy should not be interpreted as an inevitable consequence of a given OGTT‐based classification, but rather as the result of the interaction between baseline metabolic status, clinical risk factors and response to treatment during pregnancy.

The phenotypic heterogeneity of GDM, as determined by the oral glucose tolerance test, is not only a predictor for insulin therapy but is also significantly associated with adverse maternal and fetal outcomes (Chatzakis et al., 2023; Yeung et al., 2024). A meta‐analysis of eight studies (*n* = 20.928 women with GDM) evaluated pregnancy outcomes in different GDM phenotypes (Chatzakis et al., 2023). These interpretations should be considered cautiously, as OGTT‐based classifications represent operational groupings and do not directly establish distinct underlying pathophysiological mechanisms. The pooled prevalence of LGA, hypertensive disorders of pregnancy (HDP) and insulin treatment were 20%, 8% and 24%, respectively, in women with abnormal fasting plasma glucose, 10%, 6% and 9%, respectively, in women with abnormal post‐load plasma glucose, and 14%,14% and 30%, respectively, in women with abnormal combined plasma glucose. The higher risk of HDP in CH‐GDM women was likely mediated by endothelial dysfunction and pro‐inflammatory mediators (e.g., plasminogen activator inhibitor‐1 [PAI‐1], intercellular adhesion molecule‐1 [ICAM‐1], vascular cell adhesion molecule‐1 [VCAM‐1], E‐selectin), supporting that a more severe metabolic impairment is associated with worse clinical outcomes (Chatzakis et al., 2023). Similarly, the IFH‐GDM phenotype was associated with a higher risk for LGA and higher birthweight centile, consistent with the HAPO study findings (Metzger et al., 2008) and the underlying pathophysiology (Chatzakis et al., 2023).

Data from two studies (*n* = 1025) were used to assess the need of insulin treatment in pregnant women with GDM (Kotzaeridi et al., 2021; Papachatzopoulou et al., 2020). The pooled prevalence of insulin treatment was 23% (95% CI: 12%–38%, *I* ^2^: 91%) in pregnant women with abnormal fasting plasma glucose, 15% (95% CI: 9%–23%, *I* ^2^: 85%) in pregnant women with abnormal post‐load plasma glucose, and 31% (95% CI: 26%–36%, *I* ^2^: 0%) in pregnant women with abnormal combined plasma glucose. In the study of Papachatzopoulou et al. (2020), CH‐GDM was an independent risk factor for insulin treatment (OR 2.94, 95% CI: 1.93–4.47). Conversely, Kotzaeridi et al. (2021), showed that the IPH‐GDM subgroup had lower BMI at early gestation and required glucose‐lowering medications less often (28.9%) as compared to IFH‐GDM (47.8%, *P* = 0.019) and CH‐GDM (54.5%, *P* = 0.005).

These data are partially consistent with our findings, in which IPH‐GDM was significantly associated with lower odds of insulin treatment compared with IFH‐GDM, whereas CH‐GDM showed a non‐significant trend toward higher odds of insulin treatment. Even in our cohort, the IPH‐GDM subgroup required insulin treatment less often (24.5%) as compared to IFH‐GDM (42.8%) and CH‐GDM (58.6%).

Fasting plasma glucose has consistently been associated with an increased likelihood of insulin therapy in previous studies (Ford et al., 2022; Rosinha et al., 2022; Tang et al., 2019). Specifically, several investigations have identified key thresholds, ranging from 5.3 mmol/L (Ford et al., 2022) to 5.7 mmol/L (Tang et al., 2019), beyond which the probability of requiring pharmacological intervention markedly increases. Furthermore, multivariable models that combine fasting and post‐load glucose values from the 1‐ and 2‐h OGTT consistently improve predictive accuracy (Wang et al., 2021; Zaccara et al., 2023).

HbA1c has also been reported to be associated with insulin therapy requirement in several studies. Ducarme et al. (2019) reported an adjusted OR of 3.15 (95% CI: 1.03–9.69) for HbA1c as a determinant of antenatal insulin therapy, although its overall predictive accuracy remained limited (area under the curve 0.58). Several authors identified HbA1c thresholds of >5.3% (Tang et al., 2019), ≥5.4% (Weschenfelder et al., 2021) and ≥5.9% (Alunni et al., 2015) as associated with an increased likelihood of insulin requirement. Despite the consistently low sensitivity and positive predictive value across studies, HbA1c cut‐offs reliably demonstrated strong negative predictive power, particularly for ruling out the need for insulin or for discriminating between therapeutic modalities (basal vs. bolus) (Weschenfelder et al., 2021). Although HbA1c is not an independent predictor of NT failure in our cohort, we observed a trend toward higher values among women treated with insulin. Therefore, although HbA1c alone cannot be considered a definitive prognostic tool, it could contribute significantly to risk stratification and therapeutic decision‐making when evaluated in combination with other parameters.

Furthermore, our multivariable logistic regression analysis confirmed that, alongside the GDM phenotype, pre‐pregnancy BMI, timing of diagnosis, a history of spontaneous miscarriage, pregestational hypothyroidism and non‐Caucasian ethnicity were independent predictors of NT failure. Notably, several of these factors—such as obesity, early diagnosis and elevated fasting glucose—are already recognized in clinical practice as markers of higher risk. Future studies should evaluate whether multivariable models provide incremental value over clinical judgment alone. These factors can contribute to a multifactorial risk profile, suggesting that insulin requirements are influenced not only by metabolic phenotype, but also by clinical comorbidities and demographic background. Although the biological mechanisms linking abortion and GDM are not fully understood, previous studies have suggested that underlying metabolic syndromes, increased oxidative stress, inflammation or endothelial dysfunction resulting from abortions may contribute to the development of GDM in subsequent pregnancies (Vaajala et al., 2023; Wang et al., 2022).

Significant differences emerged between groups in terms of pre‐pregnancy BMI, with the insulin‐treated group showing the highest median values and the greatest prevalence of obesity. Pre‐pregnancy BMI remained independently associated with insulin therapy in the multivariable model, consistent with numerous reports in the literature (Zaccara et al., 2023). Eleftheriades et al. (2021) demonstrated that maternal BMI before conception, in conjunction with baseline fasting blood glucose, strongly influenced treatment modality, with BMI between 26 and 31 kg/m^2^ conferring a more than twofold increased likelihood of insulin therapy (OR 2.21 [95% CI: 1.42–3.43]). These findings are consistent with our results, which showed that obese women have a twofold increased risk of NT failure compared to normal‐weight women (OR 2.07 [95% CI: 1.21–3.55]).

The timing of diagnosis is also a crucial factor. We found that a diagnosis of GDM between 24 and 28 weeks of gestation was associated with a significantly reduced likelihood of insulin requirement (OR 0.45 [95% CI: 0.28–0.72]). Women screened earlier in pregnancy generally present with higher‐risk profiles, including a history of GDM, a pre‐pregnancy BMI greater than 30 kg/m^2^, or elevated first‐trimester fasting glucose levels. These factors not only result in earlier diagnosis but also predict a greater need for pharmacological intervention (Tamagawa et al., 2021).

These findings emphasize the need for individualized care strategies, incorporating both metabolic and clinical risk factors. The differentiation of GDM phenotypes has increasing clinical relevance, as it allows risk stratification of women based on the probability of dietary therapy failure and enables a more tailored monitoring strategy.

The strength of our study lies in the comprehensive evaluation of a wide range of potential predictors and the clear stratification of GDM patients according to their metabolic phenotype. Although treatment classification was based on the most intensive therapy required during pregnancy, the risk of misclassification is minimal, as escalation from insulin to NT alone is uncommon in clinical practice. We analysed multiple variables, including clinical history, anthropometric parameters and laboratory results, thereby providing a multifaceted perspective on the determinants of insulin requirement. Nevertheless, several limitations must be acknowledged.

Our analysis was primarily designed to identify factors associated with insulin requirement rather than to develop or validate a predictive model. Accordingly, variables were selected based on univariate significance and clinical considerations, and no formal assessment of model discrimination or calibration was performed. This approach may limit the predictive applicability of our findings. Future studies should focus on the development and validation of robust predictive models using appropriate methodologies, including comprehensive variable selection strategies and internal or external validation procedures.

The retrospective and monocentric design may limit the generalizability of our findings. Furthermore, we did not assess direct markers of insulin resistance (such as the HOMA index) or pancreatic secretion, which could have enriched the pathophysiological characterization of the different GDM phenotypes. Similarly, outcome data were not available for all patients.

Another limitation relates to the classification of GDM phenotypes based on OGTT values, which are known to have intra‐individual variability. Small fluctuations around diagnostic thresholds may lead to misclassification of patients across phenotypic groups. However, the use of standardized IADPSG criteria and the categorization based on clinically established cut‐offs reflect real‐world diagnostic practice. Moreover, the phenotypes were defined using combinations of glucose abnormalities rather than single isolated values, which may partially mitigate the impact of measurement variability

Another important consideration is the variability in glucose targets and insulin initiation criteria across countries and guidelines, which inherently limits comparability with other studies. Moreover, socioeconomic determinants of health were not assessed, despite their potential influence on treatment adherence, particularly regarding NT and physical activity. The degree of adherence to lifestyle recommendations in our cohort therefore remains uncertain.

Importantly, our findings should not be interpreted as suggesting that progression to insulin therapy is inevitable in women with GDM. On the contrary, early identification of women at higher risk may support more intensive lifestyle interventions, closer monitoring and optimization of GWG, with the potential to reduce the need for pharmacological treatment in selected cases.

Accordingly, the observed associations should not be interpreted as having predictive utility at the individual patient level, as no model development or validation was performed.

Future research should aim to overcome these limitations by incorporating prospective, multicentre designs, more detailed assessments of insulin sensitivity and secretion, and the inclusion of socioeconomic and lifestyle‐related factors. This will be essential to validate our findings and refine predictive models that can support a personalized, phenotype‐based approach to GDM management.

### 4.1 Conclusion

Classification of GDM into distinct phenotypes based on OGTT results is clinically relevant, as it reflects underlying metabolic profiles and is associated with insulin therapy requirement and clinical characterization of the pregnancy risks. Early identification of the phenotype at diagnosis enables a more personalized and timely therapeutic approach, particularly by recognizing women less likely to achieve glycaemic control with lifestyle interventions alone. Women with fasting abnormalities, especially when combined with post‐load glucose intolerance, represent the highest‐risk subgroup and require closer monitoring and earlier consideration of pharmacological treatment. By contrast, women with isolated postprandial hyperglycaemia often respond well to lifestyle measures, reflecting a more favourable metabolic profile.

Integrating phenotype classification with other independent predictors of insulin requirement – such as BMI, ethnicity, timing of diagnosis, medical and obstetric history – offers a pragmatic framework for individualized care. This strategy may enhance counselling, optimize resource allocation, and ultimately reduce maternal and neonatal complications.

Moving beyond a ‘one‐size‐fits‐all’ model, GDM management should evolve towards a phenotype‐based approach that informs not only tailored therapy, but also follow‐up intensity and priority of care in perinatal centres.

Further research is needed to validate this approach and to assess whether phenotype‐guided therapeutic algorithms can translate into improved perinatal outcomes. Further studies are warranted to better characterize factors associated with insulin therapy requirement and to determine whether these associations can be incorporated into validated prediction models capable of supporting individualized clinical decision‐making.

## AUTHOR CONTRIBUTIONS

Libera Troìa contributed to the study conception and design. Martina Garassino, Riccardo Bertinato, Caroline Leitao Thomaz, Giulia Turatello, Alessandro Libretti, contributed to investigation, resources, data curation. Chiara Airoldi, Alessandro Libretti contributed to formal analysis. All the authors contributed to the interpretation of data and drafting of the manuscript. The first draft of the manuscript was written by Libera Troìa Alessandro Libretti, Daniela Surico and Valentino Remorgida contributed to the manuscript critical review and editing. Libera Troìa, Daniela Surico and Valentino Remorgida supervised the work. All authors have read and approved the final version of this manuscript and agree to be accountable for all aspects of the work in ensuring that questions related to the accuracy or integrity of any part of the work are appropriately investigated and resolved. All persons designated as authors qualify for authorship, and all those who qualify for authorship are listed.

## CONFLICT OF INTEREST

None declared.

## FUNDING INFORMATION

None.

## GENERATIVE AI STATEMENT

The authors confirm that no artificial intelligence tools, including large language models (LLMs), were used in the drafting or revision of this manuscript. All content was conceived, written, and approved solely by the authors.

## Data Availability

The data presented in this study are available on request from the corresponding author due to privacy.
